# The crossroads of work and home: linkages between smoke-free policies at work and household environments

**DOI:** 10.1186/s12889-024-18658-9

**Published:** 2024-04-23

**Authors:** Amrita Gupta, Anjali Bansal, Priyanka Dixit, K. Anil Kumar

**Affiliations:** 1 School of Health Systems Studies, Tata Institute of Social Sciences, Mumbai, India; 2 International Institute for Population Sciences, Mumbai, India

**Keywords:** Second hand smoke, Smoke-free workplace, Smoke-free home, Smoke-free policies, India

## Abstract

**Background:**

Tobacco use causes over eight million global deaths annually, with seven million directly attributed to tobacco use and 1.2 million to second hand smoke (SHS). Smoke-free environments are crucial to counter SHS. Although India banned smoking in public places in 2008, SHS exposure remains high. Studies have noted that limiting smoking in workplaces, restaurants, etc., helps to reduce overall smoking and reduce SHS exposure. Under this background, the study explores the linkages between smoke-free workplaces and living in smoke-free homes in India.

**Methods:**

The two rounds of the GATS India (2009-10 and 2016-17) have been used for the study. The study focuses on male tobacco smokers working indoors or outdoors or both indoors and outdoors. The sample for the study was 2,969 for GATS 1 and 2,801 for GATS 2. Dependent variables include living in a smoke-free home, while the independent variables were adherence to a smoke-free office policy and socio-demographic variables. The two rounds of the GATS data were pooled for analysis. Statistical analysis involves bivariate and multivariate analysis.

**Results:**

Findings reveal that 41% of respondents worked in smoke-free workplaces in GATS 2. Nationally, smoke-free homes increased from 35% in 2009–2010 to 44% in 2016-17. Individuals with smoke-free workplaces were more likely to have smoke-free homes. The Southern region consistently exhibited the highest proportion of smoke-free homes. Urban areas and higher education correlated with increased smoke-free homes. Logistic regression analysis confirmed that workplace smoke-free status is a significant predictor of smoke-free homes. In GATS 2, respondents aged 30 years and above were less likely to have smoke-free homes, while education and Southern region residence positively influenced smoke-free homes.

**Conclusions:**

The correlation between smoke-free workplaces and smoke-free homes is linked to stringent workplace no-smoking policies, potentially deterring individuals from smoking at home. Opportunities exist for the expansion and stringent implementation of the smoke-free policies among Indian working adults, leveraging the workplace as a key setting for evidence-based tobacco control. The study highlights positive trends in India’s smoke-free homes, crediting workplace policies. Effective policies, education, and regional strategies can advance smoke-free homes, stressing the pivotal role of workplace policies and advocating broader implementation.

**Supplementary Information:**

The online version contains supplementary material available at 10.1186/s12889-024-18658-9.

## Background

Tobacco use, along with causing loss of lives, results in heavy social and economic costs [1]. Over eight million deaths each year are attributed to tobacco use globally, of which seven million deaths are the direct result of tobacco use, and 1.2 million deaths are due to second hand smoke (SHS) exposure among nonsmokers [2]. More than four-fifths of tobacco users globally are from low- and middle-income countries, where the burden of tobacco-related mortality and morbidity is among the highest [1]. India ranks second in both the production and consumption of tobacco worldwide [3]. There are 266.8 million current tobacco users in India aged 15 years or above. Although smokeless tobacco is the predominant form of tobacco consumed in India, 99.5 million adults aged 15 years or above currently smoke tobacco [4].

Tobacco smoking adversely affects nearly all body organs, diminishes overall general health, and often leads to incurable morbidity and mortality [5]. Smoking accounted for 200 million disability-adjusted life years (DALYs) globally in 2019 and was also identified as the major risk factor for mortality among males [5]. Apart from mortality, active smoking is associated with various morbidities, such as cardiovascular and respiratory diseases, type 2 diabetes mellitus, rheumatoid arthritis and reduced fertility among both men and women. Smoking can lead to cancer almost anywhere in the body, such as the lungs, blood, cervix, colon and rectum, esophagus, kidney, uterus, liver, oropharynx, pancreas and stomach. Smoking among women during pregnancy leads to adverse child health outcomes such as ectopic pregnancy, orofacial clefts, preterm delivery, stillbirth, low birth weight and sudden infant death syndrome [6, 7]. Maternal exposure to SHS during pregnancy is linked to unfavourable birth outcomes, including low birth weight [33–36], stillbirth [37], preterm birth [34–38], spontaneous abortion [37, 38], and birth defects [38].

Smoking, along with affecting active smokers, has detrimental health effects on those who are exposed to SHS [8, 9]. SHS exposure is defined as involuntary exposure to tobacco smoke among nonsmokers or inhaling tobacco smoke by nonsmokers against their will [9]. SHS is also called environmental tobacco smoke, involuntary smoking or passive smoking. Tobacco smoke has more than 7000 chemicals, including 250 toxins, of which 69 have the potential to cause cancer [7, 9–12]. SHS exposure is the 13th leading Level 3 risk factor for mortality, accounting for 1.30 million deaths each year globally. SHS exposure also accounts for 37.0 million DALYs, with 11.2% of the burden among children below five years [13]. SHS exposure results in a range of physical ailments, such as lung cancer and other respiratory disorders among adults and numerous ailments among children, including asthma and upper and lower respiratory tract infections [14].

A completely smoke-free environment is the only mechanism to adequately protect the health of all from the devastating effects of SHS. The advantages of smoke-free places are indubitable, and the movement to a smoke-free environment has gained momentum. In recent years, efforts have been made globally to promote smoke-free environments, including workplaces, to protect nonsmokers from the harmful effects of SHS. The “P” in MPOWER framework stands for “Protect from tobacco smoke” and emphasizes creating smoke-free environments to protect individuals from the dangers of SHS. This involves implementing laws and regulations to prohibit smoking in public places, workplaces, and other enclosed spaces to reduce exposure to SHS and protect public health [15]. A number of countries have successfully implemented the policy of smoke-free indoor workplaces and public places [16]. Considering the adverse health implications of SHS exposure, policies aimed at reducing SHS have emerged, especially for the indoor workplace. Article 8 of the WHO Framework Convention on Tobacco Control (FCTC) emphasizes the need to provide protection against tobacco smoke exposure in indoor workplaces and public places [17]. For a stringent implementation of smoking laws and legislation and to protect nonsmokers from SHS, the Government of India enacted the Cigarettes and Other Tobacco Products Act 2003 in 2004 (COTPA). Section 4 of the COTPA mandates the ban on smoking in public places, including workplaces [18]. Comprehensive smoke-free legislation not only limits the times and places for smoking but also motivates smokers to attempt to quit smoking, reduces the SHS exposure of nonsmokers and is associated with substantial health benefits for all [19–21]. In an effort to shield people from the dangers of tobacco smoke, the Indian government imposed a ban on smoking in public places and workplaces on October 2, 2008 [22].

Numerous studies have noted that policies and laws that limit smoking in workplaces, restaurants, etc., help to reduce overall smoking [23] and reduce SHS exposure among nonsmokers [24–27]. Previous studies have shown that a smoke-free workplace helps change social norms toward exposing others to SHS at home. Studies have shown that the restriction on smoking at the workplace has resulted in a ripple effect at home, resulting in the reduction of SHS exposure [20, 26, 28–32]. This linkage between smoke-free workplaces and smoke-free homes has not been much explored in low- and middle-income countries such as India, where although the rules for smoke-free public places are well documented, stringent implementation is lacking. Although there has been a decline in SHS exposure at home in India between the two rounds of the Global Adult Tobacco Survey (GATS), SHS exposure at home remains substantially high. In India, there is no legislation, in particular, for bringing down SHS exposure at home. Smoking policy at the workplace may have an effect on smoking behaviour and smoking rules at home, along with youths’ attitudes toward smoking and their smoking habits. A few studies have explored the association between smoking policies at the workplace and SHS exposure at home. However, there is a dearth of studies examining this association in the Indian context. Under the given background, the paper tries to assess the association between smoke-free workplaces and living in smoke-free homes using the GATS India data for the two time periods (2009-10 and 2016-17).

## Methods

### Data source

The two rounds of the GATS India, GATS 1 (2009-10) and GATS 2 (2016-17) are used to conduct secondary data analyses. The GATS is a nationally representative household survey of individuals aged 15 years or above. The GATS survey uses consistent and standard protocols across countries. The GATS is conducted to measure and monitor the prevalence of tobacco use, exposure to SHS, and the impact of tobacco control measures across several socio-demographic variables. The GATS is a cross-sectional, nationally representative household survey covering all states of India. A multistage sampling procedure was adopted independently in each state, and within the states, three- and two-stage sampling was used independently for urban and rural areas. The first round of GATS India was conducted in 2009-10, and the second round was conducted in 2016-17. The International Institute for Population Sciences (IIPS), Mumbai, India, and the Tata Institute of Social Sciences (TISS), Mumbai, India, were the nodal agencies for implementing GATS 1 and 2, respectively. The first round of GATS, India, was carried out in 29 states and two Union Territories (UTs), with 69,296 completed interviews of individuals aged 15 years and above. In the second round of GATS India, a total of 74,037 interviews of individuals aged 15 years and above were completed in 30 states and two UTs. Detailed methodologies of GATS 1 and 2 have been published previously [4, 33]. The study participants were current male tobacco smokers aged 15 and above who reported working indoors or both indoors and outdoors but outside their homes. For the present analysis, we have taken into consideration only the male sample, as the sample of current female smokers who were working outside homes was smaller for both rounds (for females the percentage of current smokers was 3.4% in GATS-1 (119 women), and 2.4% in GATS-2 (71 women)). The sample for the study was 2,969 for GATS 1 and 2,801 for GATS 2.

## Measures

### Dependent variables

Smoking tobacco includes products like bidi, manufactured cigarette, hand-rolled cigarette, pipe, cigar, hukkah, water-pipe, chutta, dhumti and chillum. The current tobacco smokers were defined as the person currently smoking at least one tobacco product every day over a period of one month or more. The dependent variable for the study is whether the respondents live in a smoke-free home environment, which was based on whether they reported anyone smoking inside their home in the past 30 days. The primary independent variable is whether respondents work in a strictly smoke-free office environment, as determined by the question, “Which of the following best describes the indoor smoking policy where you work: Smoking is allowed anywhere, smoking is allowed only in some indoor areas, smoking is not allowed in any indoor areas, or there is no policy?” and whether they had seen anyone smoke in indoor areas in the place where they worked in the past 30 days. Here, ‘0’ means smoking allowed in the office and observed someone smoked in the last 30 days, and ‘1’ means smoking not allowed and not seen anyone smoking. We used the following variables as covariates: age of the respondents in completed years (less than 25, 25–34, 35–44, 45–54, 55+), religion (Hindu, Muslim, Others), caste (Scheduled Caste (SC)/Scheduled Tribes (ST), Other Backward Classes (OBC), Others), place of residence (rural, urban), level of education (no education, which also includes less than primary, primary, secondary, higher than secondary), Wealth index (poorest, poorer, middle, richer, richest), type of occupation (employed, self-employed), region of residence (Central, North, East, North‒East, West, and South), and number of household members (1–4, 4+). The household wealth index was estimated using an asset index. The index was constructed based on household assets and possession of household consumer items using the principal component analysis technique. Based on time relevance, 10 and 14 household assets were included in GATS-1 and GATS-2, respectively, to create the wealth index in the respective time period. Using rank methods, households were classified by wealth quintiles.

### Statistical analysis

In this study, we pooled the two rounds of GATS: GATS-1 and GATS-2. All the dummy variables were interacted with the time period of the survey. The estimates of the different rounds of GATS are comparable because of its sampling design [34]. Studies in the past have pooled different GATS rounds to examine trends over time [35, 36]. To measure the effect of the office-level policy on home smoke-free environment over the time period, we fitted a pooled binary logistic regression analysis. In the pooled binary logistic regression model, the interaction between time of the survey and all the predictor variables were created, and the results of this analysis are presented as a set of predicted probabilities to see the changes in the smoke-free home environment because of the smoke-free office environment in both rounds of GATS after adjusting all the socio-economic and demographic variables. The advantage of using the binary logistic regression procedure is that it models the log of the odds of an outcome occurring in terms of a vector of independent variables. The individual-level associations between the respondents working in a smoke-free office environment and its effect on the smoke-free home environment at the national level were examined using bivariate and multivariable logistic regression. A bivariate analysis was performed and manifested the mean smoke-free status of the workplace on home for all states in India for GATS-1 and GATS-2. The adjusted odds ratio (AOR) along with the 95% confidence interval (CI) were calculated for respondents who worked in a smoke-free environment compared with those who were not working in a smoke environment.

## Results

A summary description of all the covariates is presented in Table S1. 41% of the respondents worked in a smoke-free workplace in GATS 2. The respondents were mainly from rural areas, in the age group 30–44 years and belonged to the Hindu religion. Table 1 presents the percentage distribution of smoke-free homes by various covariates. At the national level, 35% of the homes were smoke-free during 2009–2010, which increased to 44% by 2016-17. 49% of the respondents in GATS 1 who worked in a smoke-free workplace also had a smoke-free home. In GATS 2, this figure increased by 10% points. 59% of the respondents working in smoke-free workplaces also had smoke-free homes in GATS 2. On the other hand, among the respondents who did not work in a smoke-free workplace, the percentage of smoke-free homes was only 25.6% and 32.5% in GATS 1 and GATS 2, respectively. In GATS 2, the Southern region had the highest proportion of smoke-free homes (75.1%), followed by the Western region (55%). A similar picture was observed for the smoke-free homes by region in GATS 1. Urban areas had more smoke-free homes than rural areas. In urban areas, 43% and 53% of the respondents living in smoke-free homes in GATS 1 and GATS 2, respectively. For different social strata, it was found that a higher proportion of respondents belonging to the OBC group (49.7%) had smoke-free homes compared to the other caste groups. Respondents belonging to the Hindu religion (46.0%) had the highest share of smoke-free homes, followed by the ‘other’ religious group (41.8%) and Muslims (34.9%). The educational status of the respondent also impacted the smoke-free environment at home. A higher percentage of respondents with higher than secondary education lived in smoke-free homes compared to the other educational groups (42.1% GATS 1; 66.2% GATS 2). A higher percentage of respondents belonging to the richest wealth quintile households lived in smoke-free homes compared to their counterparts (51.4% in GATS-1; 59.6% in GATS-2). Households with four or fewer members had a higher percentage of smoke-free homes compared to respondents with more than four household members (41.7% GATS 1; 50.2% GATS 2).

**Table 1:** Bivariate distribution for smoke-free environment at home by selected covariates, GATS, India 2009-17

Background characteristics	GATS 1	GATS-2
**Smoke free status of workplace**		
No	25.6	32.5
Yes	49.4	58.7
**Age of respondents**		
15–29	36.9	49.5
30–44	37.8	42.3
45+	31.5	41.9
**Religion**		
Hindu	NA	46.0
Muslim	NA	34.9
Others	NA	41.8
**Caste**		
SC/ST	NA	37.9
OBC	NA	49.7
Others	NA	40.5
**Place of residence**		
Urban	43.0	53.3
Rural	29.3	38.0
**Educational status**		
No education	29.4	35.0
Primary	35.6	46.1
Secondary	39.0	45.4
Higher than secondary	42.1	66.2
**Wealth Index**		
Poorest	27.2	28.5
Poorer	35.6	40.2
Middle	34.1	46.8
Richer	41.6	54.3
Richest	51.4	59.6
**Occupation**		
Employed	36.6	43.4
Self-employed	34.1	44.1
**Region**		
North	16.1	26.5
Central	27.3	37.3
East	30.4	39.4
North‒East	24.3	35.4
West	41.4	54.9
Southern	56.0	75.1
**Number of household members**		
1–4	41.7	50.2
4+	31.5	38.7
**Total**	**35.4**	**43.9**

Note: In GATS-1, data on caste and religion were not available

Table 2 presents the AOR and 95% CI to find the association of a smoke-free home environment with different covariates and no-smoke policy at the workplace. Smoke-free status of the workplace emerged as a significant predictor of smoke-free homes in both GATS 1 and 2. In GATS 1, apart from a smoke-free workplace, place of residence and region of residence were significant predictors of smoke-free homes. In GATS 2, educational status and region of residence were significant predictors of a smoke-free home environment. At the national level, if the respondents had a smoke-free environment at the workplace, they were more likely to have a smoke-free home environment in GATS 1 and GATS 2 (GATS 1 AOR = 2.63 95% CI (1.85–3.73); GATS 2 AOR = 2.51 95% CI (1.87–3.38)). In GATS 2, the respondents aged 30 years and above were significantly less likely to stay in smoke home-free homes than those aged 15–29 years (30–44 years AOR = 0.6, 95% CI (0.41–0.91); 45 + years AOR = 0.59, 95% CI (0.38–0.92)). Educated respondents were more likely to have a smoke-free home environment than those with no education in GATS 2. Respondents with higher than secondary education were 2.1 times more likely to live in smoke-free homes than those with no education in GATS 2 (AOR = 2.1, 95% CI (1.24–3.57). The respondents from the Southern region were more likely to have a smoke-free home environment than those from the Northern region (GATS 1 AOR = 7.00, 95% CI (4.21–11.61); GATS 2 AOR = 8.49, 95% CI (5.52–13.07)). However, there was no significant relationship between the types of occupation and smoke-free homes for either round of GATS.

**Table 2:** Adjusted odds ratio along with 95% CI to examine the effect of different covariates on smoke-free homes in India, GATS, India 2009-17

Background characteristics	GATS 1	GATS 2
**Smoke free status of workplace**		
No		1.00
Yes	2.63***(1.85 3.73)	2.51***(1.87 3.38)
**Age of respondents**		
15–29	1.00	1.00
30–44	0.89 (0.54 1.47)	0.61**(0.41 0.91)
45+	0.62 (0.36 1.05)	0.59**(0.38 0.92)
**Religion**		
Hindu		1.00
Muslim		0.65 (0.4 1.04)
Others		1.05 (0.61 1.81)
**Caste**		
SC/ST		1.00
OBC		1.44 (0.99 2.09)
Others		1.23 (0.78 1.95)
**Place of residence**		
Urban		1.00
Rural	0.67**(0.48 0.94)	0.74 (0.53 1.03)
**Educational status**		
No education	1.00	1.00
Primary	1.15 (0.75 1.78)	1.54**(1.08 2.21)
Secondary	1.25 (0.73 2.15)	1.24 (0.81 1.88)
Higher than secondary	1.17 (0.66 2.07)	2.1***(1.24 3.57)
**Wealth Index**		
Poorest	1.00	1.00
Poorer	1.47** (1.07 2.04)	1.25 (0.94 1.66)
Middle	1.54** (1.01 2.18)	1.44**(1.09 1.90)
Richer	1.48** (1.05 2.07)	1.71***(1.25 2.34)
Richest	2.15***(1.51 3.07)	1.67***(1.20 2.32)
**Occupation**		
Employed	1.00	1.00
Self-employed	1.25 (0.89 1.75)	0.95 (0.68 1.32)
**Regions**		
North	1.00	1.00
Central	2.63***(1.38 5)	1.99***(1.26 3.13)
East	2.81***(1.66 4.77)	2.04***(1.31 3.17)
North‒East	1.94***(1.22 3.09)	1.64**(1.09 2.47)
West	3.68***(2.11 6.42)	2.83**(1.24 6.45)
Southern	7.00***(4.21 11.61)	8.49***(5.52 13.07)
**Number of household members**		
1–4	1.00	1.00
4+	0.85 (0.62 1.17)	0.91 (0.68 1.21)

*p < 0.1; **p < 0.05; ***p < 0.000

**Fig. 1:**
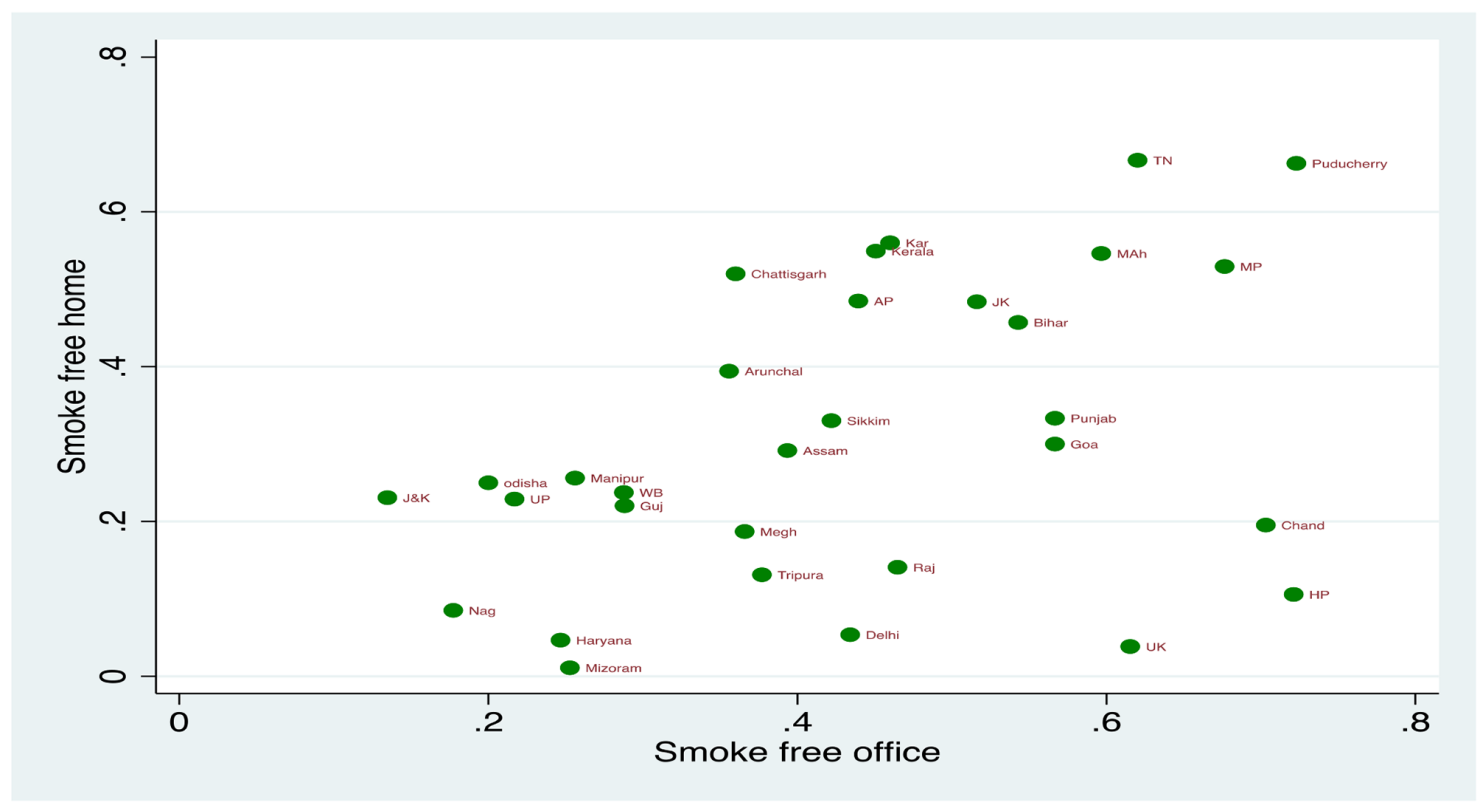
State-level association between smoke-free environment at work and smoke-free homes for states of India, GATS 1. Note: J&K-Jammu & Kashmir, HP-Himachal Pradesh, PB-Punjab, UK-Uttarakhand, HR- Haryana, RAJ-Rajasthan, UP-Uttar Pradesh, MP-Madhya Pradesh, WB- West Bengal, JH- Jharkhand, BR- Bihar, NAG- Nagaland, MEG- Meghalaya, GUJ- Gujarat, MAH- Maharashtra, AP-Andhra Pradesh, KA- Karnataka, TN- Tamil Nadu. Correlation coefficient (rs)-0.41; *p* < 0.020

**Fig. 2:**
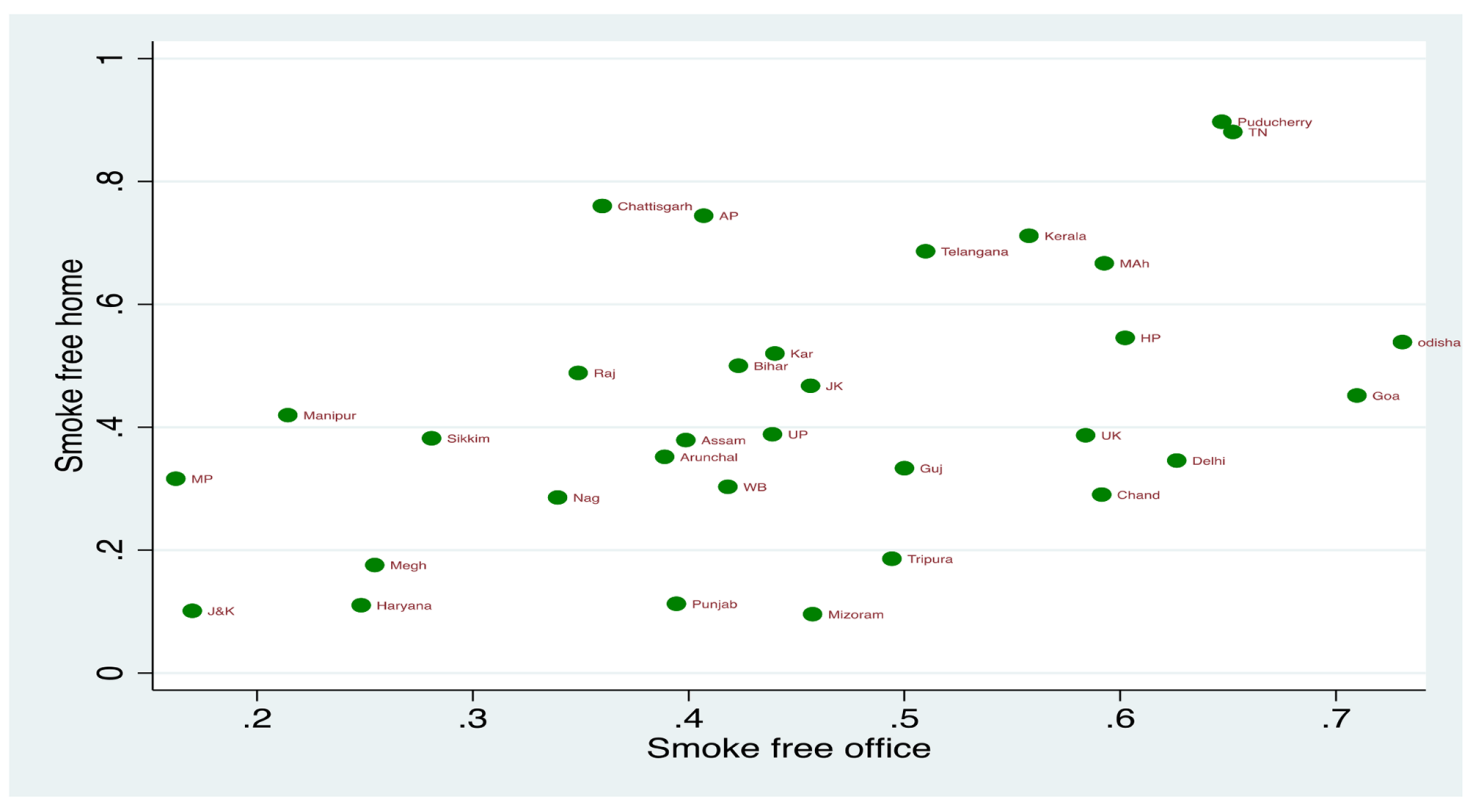
State-level association between smoke-free environment at work and smoke-free homes for states of India, GATS 2. Note- J&K-Jammu & Kashmir, HP-Himachal Pradesh, PB-Punjab, UK-Uttarakhand, HR- Haryana, RAJ-Rajasthan, UP-Uttar Pradesh, MP-Madhya Pradesh, WB- West Bengal, JH- Jharkhand, BR- Bihar, NAG- Nagaland, MEG- Meghalaya, GUJ- Gujarat, MAH- Maharashtra, AP-Andhra Pradesh, KA- Karnataka, TN- Tamil Nadu. Correlation coefficient (r_s_) = 0.48; *p* < 0.000

**Fig. 3:**
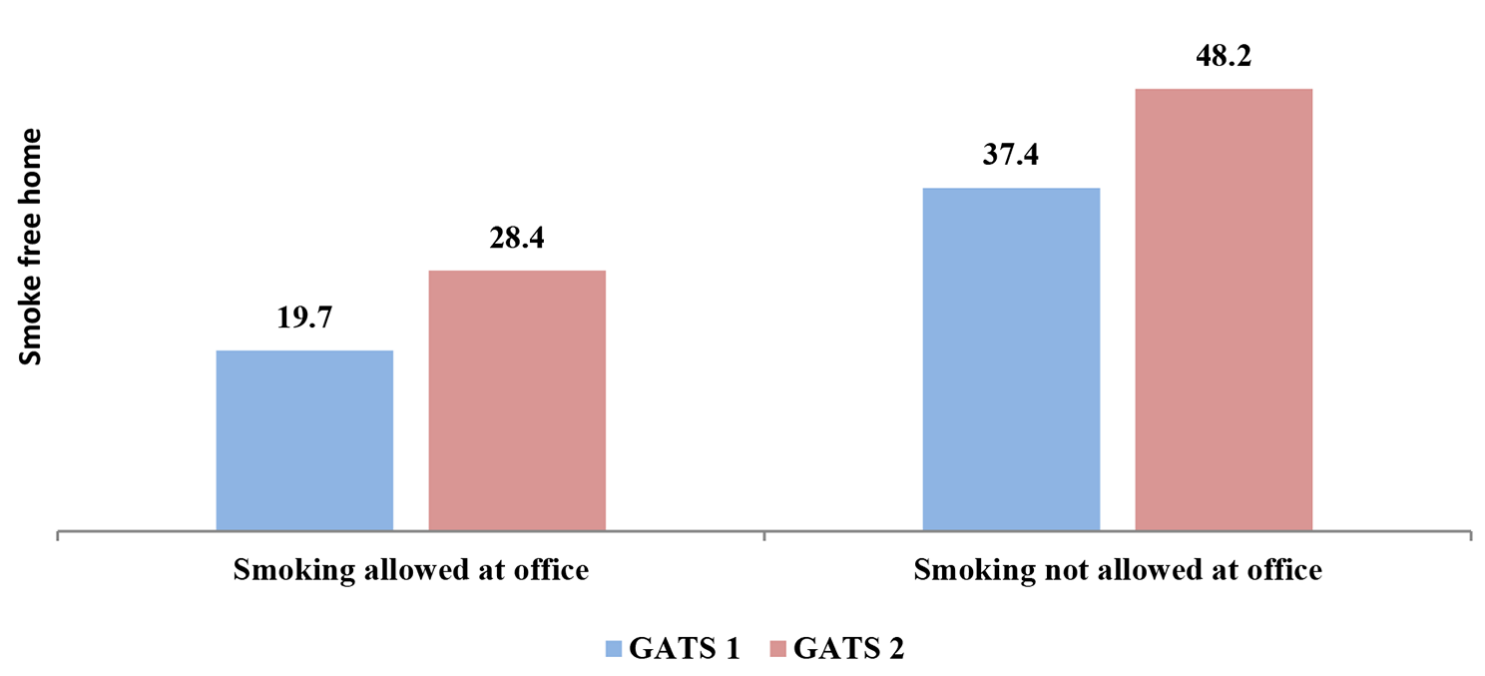
Mean predicted probability of smoke-free homes by smoke exposure at workplace, GATS 1 and GATS 2. Note: adjusted for all the covariates

Figures 1 and 2 present the mean effect of the smoke-free office environment on the smoke-free home environment among different states of India for GATS 1 and GATS 2. The states with a higher percentage of smoke-free workplaces had a higher percentage of smoke-free homes (GATS 1 r_s_-0.41; *p* < 0.020; GATS 2 r_s_=0.48; *p* < 0.000). Figure 3 shows the mean predicted probabilities of smoke-free homes in GATS 1 and GATS 2 by office smoke exposure. It was found that smoke-free homes increased significantly from GATS 1 to GATS 2. By the smoking exposure at office also, there is an increase in smoke-free homes, i.e. compared to GATS 1, there is a significant increase in smoke-free homes in GATS 2 due to the smoke-free office environment. In GATS-2, 59% of homes are smoke free when smoking not allowed at workplace, which has increased from 46% in GATS-1.

## Discussion

The present study explores the association between smoke-free policies at the workplace and smoke-free environment at home utilizing the data from the two rounds of the GATS India. The study findings show that a higher proportion of individuals with smoke-free workplaces had smoke-free homes for both rounds of the survey. Encouragingly, the percentage of smoke-free homes in India has increased by 8.5% points between GATS 1 (35.4%) and GATS 2 (43.9%). A higher proportion of individuals from smoke-free workplaces had smoke-free homes in GATS 1 (49.4%) and GATS 2 (58.7%). Sixty-four percent of adults with smoke-free workplaces had smoke-free homes compared with only 42% of adults who were exposed to SHS at work. In line with the study findings, previous studies have also conveyed a significant association between the no smoking policy at the workplace and smoke-free homes [31, 32, 37–40].

A higher proportion of individuals from the Hindu religion and OBC caste group had smoke-free homes compared to their counterparts in GATS 2. Urban areas exhibited a higher prevalence of smoke-free homes than rural areas for both the rounds of GATS. There were variations in the smoke-free homes by the regions, with the Southern region having the highest prevalence for both GATS 1 (56.0%) and GATS 2 (75.1%). Higher than secondary education and lower household size were associated with a higher percentage of smoke-free homes for both GATS 1 and GATS 2. The smoke-free home environment was higher for respondents working in the formal sector. However, a study in Ireland reported higher smoking rates among those working in the informal sector, primarily due to difficult and enduring economic circumstances [41]. The binary regression results highlight that smoke-free environments at the office significantly increase the likelihood of a smoke-free home environment. Education was a significant predictor of smoke-free homes in India, with educated people being more likely to have a smoke-free home environment than uneducated respondents. States such as Telangana and Tamil Nadu were more likely to have smoke-free home environment if they had smoke-free offices. Previous studies have documented the varying levels of tobacco use across Indian states attributable to the social environment and other contextual factors, such as cultural and social norms and the implementation of tobacco control policies in a given area [42–45]. Smoke-free workplaces, place of residence and region were significant predictors of smoke-free homes in GATS 1. In GATS 2, smoke-free workplace status, age, educational status, and region were significant predictors of smoke-free homes. At the national level, respondents with smoke-free workplaces were 2.63 and 2.51 times more likely to have smoke-free homes in GATS 1 and GATS 2, respectively. In GATS 2, education played a significant role, with higher education positively influencing smoke-free homes. A higher proportion of individuals from higher wealth quintile households had smoke-free homes compared tothan respondents from lower wealth quintile households. Compared to the Northern region, all the other regions had higher odds of smoke-free homes for both rounds of the GATS. However, occupation type showed no significant relationship with smoke-free homes in either survey. There was a significant positive relationship among individuals working in a smoke-free workplace and having a smoke-free home across the states/UTs for both rounds of GATS in India. The study indicates positive growth in smoke-free homes in India, with workplace policies playing a pivotal role.

## Conclusion

The findings presented in the study shed light on the dynamic interplay between workplace smoking policies and the prevalence of smoke-free environments in homes across regions and social strata in India. The study findings offer valuable insights into the progress made in establishing smoke-free homes and the factors influencing this trend over two rounds of the GATS India data. Notably, there has been a noticeable increase in the percentage of smoke-free homes nationally from 2009 to 2010 to 2016-17, signifying a positive shift in societal attitudes towards smoke-free environments. The correlation between having a smoke-free workplace and a smoke-free home is strong, with the likelihood of having a smoke-free home being significantly higher for those employed in smoke-free workplaces. The findings highlight the cascading effect of workplace policies on societal norms and behaviours related to tobacco use at home. The association between smoke-free workplace and smoke-free home can be attributed to the strict no-smoking policy at the workplace, which may restrict an individual from smoking at home and eventually lead to quitting. Studies have also found that smoke-free workplaces help employees reduce and even discontinue tobacco use [46–50]. Opportunities exist to increase the amount of smoke-free policy coverage among Indian working adults, as the workplace can serve as a significant setting for the promotion of evidence-based tobacco control and prevention strategies [51].

The study underscores the progress in achieving smoke-free homes in India and identifies areas for targeted interventions. The findings from this study present compelling evidence that reinforces the urgent need for stricter enforcement of the ban on smoking in workplaces in India, which in turn would improve the smoke-free home environment. India has undertaken several steps to control tobacco consumption and restrict tobacco use in workplaces, which have evolved and expanded across the country [52]. Smoke-free laws and the denormalization of smoking are vital tools for tobacco control [53]. The smoke-free policies help in reducing a range of adverse health outcomes [54–56]. Studies also indicate that a smoking ban directly influences a range of smoking behavior and promotes quitting or reducing tobacco consumption [46, 57, 58].

To strengthen the workplace smoke-free policy, there should be stringent implementation of the smoke-free policies across all sectors. Government agencies and private companies should implement and rigorously enforce policies that prohibit smoking in indoor work areas. Additionally, awareness campaigns should be carried out in the workplace to educate individuals about the benefits of smoke-free environments, both in the workplace and at home. These campaigns should emphasize the health risks associated with tobacco use and SHS exposure and highlight the positive impact of smoke-free policies on reducing these risks. Recognizing the disparities in smoke-free home environments across different regions and socioeconomic and demographic groups the targeted interventions need to be developed to address the barriers to adopting smoke-free policies belonging to the high focused groups. This could include tailored educational programs, subsidies for smoke-free initiatives, or community-based support networks. Policymakers should also implement region-specific strategies to promote smoke-free policies. This might include targeted outreach efforts, incentives for smoke-free initiatives, and collaboration with local authorities and community organizations. There should be robust monitoring and evaluation mechanisms, such as surveys at regular intervals, to track the implementation and effectiveness of smoke-free policies over time. The data-driven approach can inform future policy decisions and resource allocation. Government should invest in capacity-building initiatives to support organizations, communities, and individuals in adopting and maintaining smoke-free policies. This could involve providing technical assistance, training programs, and resources to help stakeholders implement and sustain smoke-free environments. There should be steps to integrate efforts to promote smoke-free environments with broader tobacco control initiatives, including measures to reduce tobacco consumption, prevent youth initiation, and support smoking cessation. By addressing both the supply and demand sides of tobacco use, policymakers can create synergistic effects and maximize the impact of their interventions. Overall, a comprehensive approach that combines policy interventions, public awareness campaigns, targeted interventions, and regional strategies is essential to creating and sustaining smoke-free environments in workplaces and homes. By prioritizing these recommendations, policymakers can protect the health and well-being of individuals, families, and communities across India.

### Electronic supplementary material

Below is the link to the electronic supplementary material.

Supplementary Material 1

## Data Availability

No datasets were generated or analysed during the current study.

## Abbreviations

AOR: Adjusted odds ratio
COTPA: Cigarettes and Other Tobacco Products Act 2003 in 2004
CI: Confidence interval
DALYs: Disability-adjusted life years
FCTC: Framework Convention on Tobacco Control
GATS: Global Adult Tobacco Survey
IIPS: International Institute for Population Sciences
SHS: Secondhand smoke
TISS: Tata Institute of Social Sciences
UTs: Union Territories
