# Supplementary material for: The crossroads of work and home: linkages between smoke-free policies at work and household environments
Source: BMC Public Health. 2024 Apr 23;24:1127. doi: 10.1186/s12889-024-18658-9 (PMC11040815; doi:10.1186/s12889-024-18658-9)
Supplement: Supplementary file 1 — Supplementary Material 1 [file 12889_2024_18658_MOESM1_ESM.docx]

**Table S1: Sample Description, GATS, 2009-10 and 2016-17, India**

| **Background characteristics** | **GATS 1** | | **GATS 2** | |
| --- | --- | --- | --- | --- |
|  | **Number** | **Percentage** | **Number** | **Percentage** |
| **Smoke exposure at home** |  |  |  |  |
| Smoke allowed at home | 2,145 | 64.6 | 1,766 | 56.1 |
| Smoke not allowed | 796 | 35.4 | 1,035 | 43.9 |
| **Smoke free status of workplace** |  |  |  |  |
| No | 1,723 | 58.5 | 1,644 | 58.7 |
| Yes | 1,218 | 41.5 | 1,157 | 41.3 |
| **Age of respondents** |  |  |  |  |
| 15-29 | 465 | 19.0 | 487 | 20.6 |
| 30-44 | 1,535 | 46.3 | 1,320 | 41.1 |
| 45+ | 941 | 34.8 | 994 | 38.3 |
| **Religion** |  |  |  |  |
| Hindu | NA | NA | 1,889 | 75.8 |
| Muslim | NA | NA | 441 | 20.1 |
| Others | NA | NA | 471 | 4.1 |
| **Caste** |  |  |  |  |
| SC/ST | NA | NA | 1,055 | 27.7 |
| OBC | NA | NA | 881 | 41.8 |
| Others | NA | NA | 865 | 30.5 |
| **Place of residence** |  |  |  |  |
| Urban | 1,430 | 45.0 | 1,017 | 36.7 |
| Rural | 1,511 | 55.0 | 1,784 | 63.3 |
| **Educational status** |  |  |  |  |
| No education | 663 | 29.9 | 831 | 36.2 |
| Primary | 890 | 29.9 | 955 | 33.6 |
| Secondary | 838 | 23.6 | 712 | 21.9 |
| Higher than secondary | 550 | 13.8 | 303 | 8.4 |
| **Wealth Index** |  |  |  |  |
| Poorest | 404 | 21.5 | 570 | 25.8 |
| Poorer | 504 | 21.7 | 511 | 15.6 |
| Middle | 594 | 17.8 | 611 | 23.7 |
| Richer | 631 | 19.2 | 462 | 16.3 |
| Richest | 808 | 19.8 | 647 | 18.5 |
| **Occupation** |  |  |  |  |
| Employed | 1,650 | 53.8 | 1,882 | 66.4 |
| Self Employed | 1,291 | 46.2 | 919 | 33.6 |
| **Region** |  |  |  |  |
| North | 900 | 14.5 | 920 | 20.5 |
| Central | 142 | 16.4 | 276 | 27.8 |
| East | 365 | 24.4 | 287 | 20.4 |
| North‒East | 927 | 6.5 | 851 | 7.2 |
| West | 208 | 12.0 | 78 | 4.4 |
| South | 399 | 26.2 | 389 | 19.8 |
| **Number of household members** |  |  |  |  |
| 1-4 | 1,332 | 38.7 | 1,473 | 52.6 |
| 4+ | 1,609 | 61.3 | 1,328 | 47.4 |
| **Total** | **2,941** | **100.0** | **2,801** | **100.0** |

Note: Number of cases are unweighted. Percentages are weighted
